# A computational framework for canonical holistic morphometric analysis of trabecular bone

**DOI:** 10.1038/s41598-022-09063-6

**Published:** 2022-03-25

**Authors:** Sebastian Bachmann, Christopher J. Dunmore, Matthew M. Skinner, Dieter H. Pahr, Alexander Synek

**Affiliations:** 1 Institute of Lightweight Design and Structural Biomechanics, TU Wien, Vienna, Austria; 2 School of Anthropology and Conservation, Skeletal Biology Research Centre, University of Kent, Canterbury, UK; 3 Department of Human Evolution, Max Planck Institute for Evolutionary Anthropology, Leipzig, Germany; 4 Department of Anatomy and Biomechanics, Division Biomechanics, Karl Landsteiner University of Health Sciences, Krems, Austria

**Keywords:** Anthropology, Image processing, Biomedical engineering, Bone

## Abstract

Bone is a remarkable, living tissue that functionally adapts to external loading. Therefore, bone shape and internal structure carry information relevant to many disciplines, including medicine, forensic science, and anthropology. However, morphometric comparisons of homologous regions across different individuals or groups are still challenging. In this study, two methods were combined to quantify such differences: (1) Holistic morphometric analysis (HMA) was used to quantify morphometric values in each bone, (2) which could then be mapped to a volumetric mesh of a canonical bone created by a statistical free-form deformation model (SDM). Required parameters for this canonical holistic morphometric analysis (cHMA) method were identified and the robustness of the method was evaluated. The robustness studies showed that the SDM converged after one to two iterations, had only a marginal bias towards the chosen starting image, and could handle large shape differences seen in bones of different species. Case studies were performed on metacarpal bones and proximal femora of different primate species to confirm prior study results. The differences between species could be visualised and statistically analysed in both case studies. cHMA provides a framework for performing quantitative comparisons of different morphometric quantities across individuals or groups. These comparisons facilitate investigation of the relationship between spatial morphometric variations and function or pathology, or both.

## Introduction

Living bone can functionally adapt^1^ to loads it experiences and thus reflects, to some extent, the behaviour of individuals over their lifetime. This functional adaptation, caused by modelling and remodelling of bone^2^, has been experimentally demonstrated in external shape change^1,3^, the thickening of cortical bone^1,4^, entheseal shape change on the cortical bone surface^5,6^, and change in the architecture of trabecular bone^7,8^, or a combination of these phenomena. Unlike cortical bone, trabecular architecture is usually completely internal, concentrated in the epiphyses of long bones, and far more porous at the mesoscale^9^. As a result of these and many other differences, trabecular bone reacts to experienced loads differently than cortical bone, and thus records different information about these loads. Inference of function from trabecular bone architecture is complicated by genetic, ontogenetic^10^, and systemic^11^ factors that also influence bone form. However, numerous studies have found trabecular architectures that reflect assumed habitually and significantly loaded joint postures^12–24^, regardless of whether this load is gravitational, muscular, or a combination of both^25,26^. Thus, analysis of trabecular architecture provides additional information that can be combined with studies of internal and external cortical bone morphology, to provide a more holistic understanding of how bone reflects behaviour. We can use the bone morphology of recent species, with observable behaviours, to infer behaviour patterns of extinct species^27^. Therefore, methods to evaluate the trabecular morphologies of different species and compare them, offer an important tool to help answer evolutionary biological or anthropological questions.

Many different protocols exist to quantify the inner, trabecular structure of bone. These range from quantitative computed tomography (QCT) with a coarse resolution in the range of half a millimetre to high resolution techniques such as micro-computed tomography ($\upmu$CT) with resolutions in the micrometre range. Using QCT it is possible to get estimates of bone-mineral density or bone volume fraction (BVTV), however other morphometric quantities can only be assessed at a high resolution, which is able to depict the trabecular structure in detail. $\upmu$CT allows for the imaging of the complete trabecular structure, and a variety of CT-based morphometric quantities exist to describe the bony network^28^. Two parameters, namely BVTV and local anisotropy, measured via a fabric tensor, are of special interest. BVTV alone accounts for approximately 87% and both values together for 97% of the trabecular stiffness, which is a proxy for the mechanical properties of the bone^29^.

Morphometric quantities can be measured for the whole trabecular volume, which yields a single value which can be compared between samples. However, the trabecular structure can be very heterogeneous—for example at the proximal femur—and thus it is not possible to gather precise differences using summary statistics on this multi-modal distribution. Therefore, a common approach is the use of regions of interest (ROI), where a subsection of the trabecular volume is selected for the investigation. One or multiple, spherical, cubic or arbitrarily shaped ROIs can be used. However, the placement of the ROI inside the bone at a homologous, i.e., anatomically equivalent, location is crucial when comparing different individuals and can be challenging in species with disparate morphology^30^. To ameliorate such problems, multiple ROIs^31,32^ or sectors^33–36^, or the combination of both^37^ can be used.

Another method that avoids many of these issues, is holistic morphometric analysis (HMA)^12,38^. HMA can map the morphometric quantities continuously over the whole trabecular volume onto volume meshes, which can then be visualised and compared qualitatively between different samples without ROI selection. It was successfully applied to a variety of bones, including carpals, metacarpals and phalanges^12–16,36^, proximal femora^17–19^, distal femora^20^, distal tibiae^17^, proximal humeri^17^, distal radii^21^, and first metatarsals^22^. However, HMA is not yet able to compare the site-specific morphometry, below the sector level, for different individuals quantitatively in homologous regions^23,24,27^.

The HMA method has also been successfully applied in combination with other methods. For example a sector-based analysis^23,36^ can be employed, but, as with other sector methods, requires a-priori geometric division of the trabecular structure. Trabecular mapping^39^ offers another way, where morphometric quantities are measured below the cortical bone and mapped onto the periosteal surface. A similar approach was applied to map HMA morphometric quantities to the endosteal surface of metacarpals^15,16^. However, both of these approaches are necessarily limited to analyse the edge of the trabecular network. Volumetric sliding semi-landmarks^40^ and coherent point drift^41^ allow for volumetric quantitative comparisons between samples using HMA^24^.

The principle in these methods is to find a canonical representation, either a surface or volume, on which all samples can be mapped, measured and finally compared. A volumetric canonical bone can also be created using statistical deformation models (SDM)^42^. The individual bones can then be registered onto the canonical bone and isotopological meshes can be created using mesh-morphing^43^. The advantage of the SDM approach is that it is landmark-free, and thus requires no extra annotation of the data. However, currently SDM methods have only been applied to QCT data, whereas $\upmu$CT data is required in order to evaluate CT-based morphometric quantities. Furthermore, SDM approaches have, so far, only been used to create canonical models in a single species, which often have less inter-subject variation in bone shape.

The aim of this work was to develop a workflow for quantitative volumetric comparison of bone morphometry at homologous sites directly using $\upmu$CT images and representing bones of different species, without the need for landmarking nor definition of ROIs or subregions. The workflow consists of the canonical bone creation using SDM, registration of individual bones, mesh morphing and finally the application of HMA and statistical analysis. We refer to this workflow as canonical holistic morphometric analysis (cHMA). The objectives of this study were: (1) to identify cHMA method parameters suitable for the analysis of $\upmu$CT images and comparison of different species, (2) to investigate the robustness of the cHMA method, and (3) to test the ability of cHMA to replicate previously published morphometric comparisons of metacarpals and femora of different primate taxa in two case studies.

## Materials and methods

### HMA method

**Figure 1:**
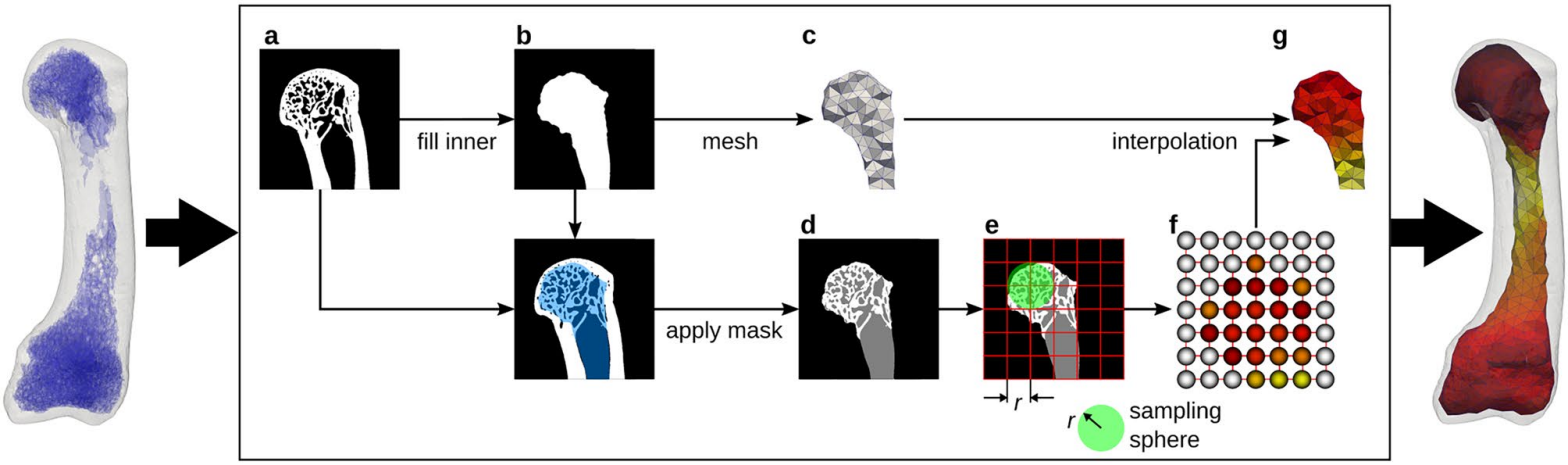
Mapping of morphometric quantities of trabecular bone onto finite-element meshes using the HMA method. The schematic overview shows the required image processing steps for a 2D slice, although the principle is similar in 3D. Original segmented $\upmu$CT image (**a**). Trabecular mask (**b**). Mesh generated from trabecular mask (**c**). Masked trabecular volume (**d**). Evaluation of morphometric quantity using background grid with grid distance *r* and sampling sphere with radius *r* (**e**). Evaluated quantity at the background grid vertices (spheres not to scale) (**f**). Linear interpolated quantity on the mesh (**g**).

The HMA method is the basis for the new cHMA workflow and is described in detail by Gross et al.^38^ and Tsegai et al.^12^. Therefore only a brief outline shall be given here; the workflow is shown graphically in Fig. 1. HMA can map a wide variety of morphometric quantities of bones^28^ from both trabecular and cortical volumes onto finite element meshes. Binary segmented $\upmu$CT images, depicting the microstructure of the bone, are used as the input for HMA. The first step of the process is to separate the trabecular volume from the whole volume using the “fill” method^44^. This method creates two image masks for the bone: a cortical and a trabecular mask. Separate masks are required to measure morphometric quantities unique to either the cortical or the trabecular bone. Then a volumetric mesh is created for the cortical and the trabecular volume, respectively. In the next step a regular grid, the so called background grid, is created and morphometric quantities are evaluated in a spherical ROI centred at each grid vertex. The sphere has the same radius as the spacing of the grid, thus the sampling spheres overlap. Typically, 2.5 mm grid spacing and 5 mm sphere diameter are used due to the underlying homogenisation theory^45,46^. In the last step, the measured values at the background grid vertices are interpolated tri-linearly at the centroids of the mesh elements.

### The cHMA method

#### Outline

**Figure 2:**
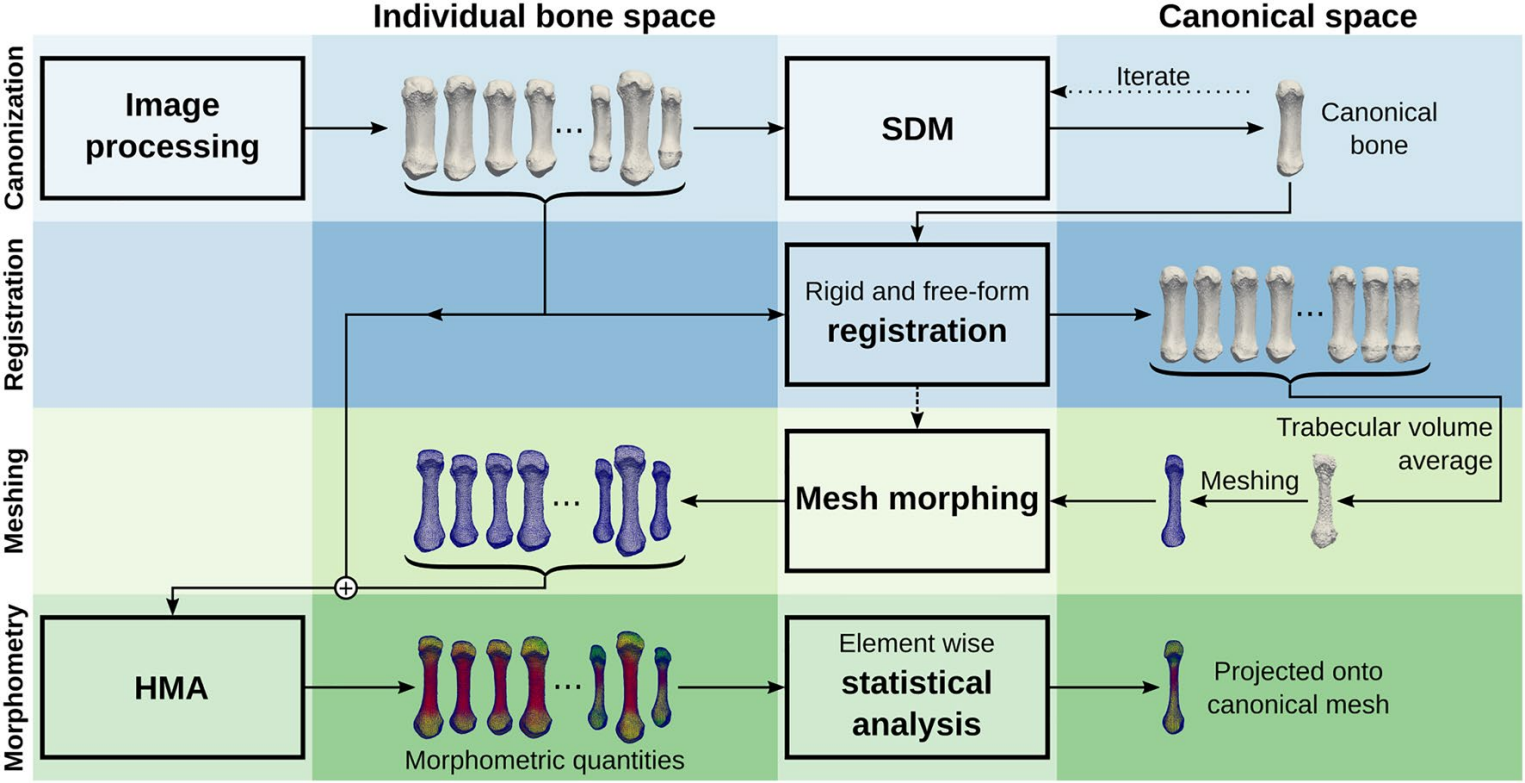
Graphical summary. Each block describes a separate step in the workflow, which runs from top to bottom.

The cHMA method extends the established HMA workflow and combines it with several other methods. A graphical summary can be seen in Fig. 2. It works by creating a canonical reference onto which different HMA results can be mapped and thus directly compared. The first stage of the workflow is to create a canonical bone image by using a statistical deformation model (SDM). In the next stage, all individual bone images are registered onto the canonical bone image, yielding transformations that map each bone volume onto the canonical bone image. The trabecular volume within all individual bone images is transformed into the canonical space, averaged and meshed using a tetrahedral mesher, which is then referred to as the canonical mesh. Isotopological meshes are created by morphing the canonical mesh back into each individual bone space. The HMA method can then be applied using the original bone images and these individual meshes, which possess homologous elements across the sample. In a further step, the mapped morphometric quantities can be statistically compared at each element across the samples. If required, results of HMA or the statistical analysis can be mapped back onto the canonical mesh and evaluated further.

#### Statistical deformation model

The SDM is created similar to Steiner et al.^47^ but the process shall be repeated here briefly. The method operates on masked images of bones, where one image (the mask) describes the label of each voxel (bone or background) while the other image contains the actual grey-value. These can be the same images as required for the HMA workflow. All images are registered onto a randomly chosen reference image using a similarity transform. This type of transform allows only for translation, rotation and isotropic scaling. All similarity transformations are averaged by taking the arithmetic mean of the translation and scaling as well as averaging the rotation by quaternion averaging^48^. The centre of rotation is fixed in all transformations. The inverse of the averaged transformation is then applied to the reference image, to scale, rotate and translate the reference image into an average position. In the next step all images are registered onto the aligned reference image. First, a similarity transform is again used, then a free-form deformation is applied using a cubic B-spline transformation. Such a B-spline transformation is parametrized using a so called control point grid, which is deformed to create the overall image transformation. When all images are registered, the transformed images and the B-spline control point grid displacements are averaged. The resulting average B-spline transformation is then inverted and applied to the averaged image, yielding the new reference image. The steps of registering and averaging can be iterated several times, until the model has converged. Diminishingly qualitative changes in the canonical bone shape indicate the convergence of the model, while the surface distance between two consecutive iterations can be used for quantification. The last reference image is then used as the canonical bone image for the further steps.

**Figure 3:**
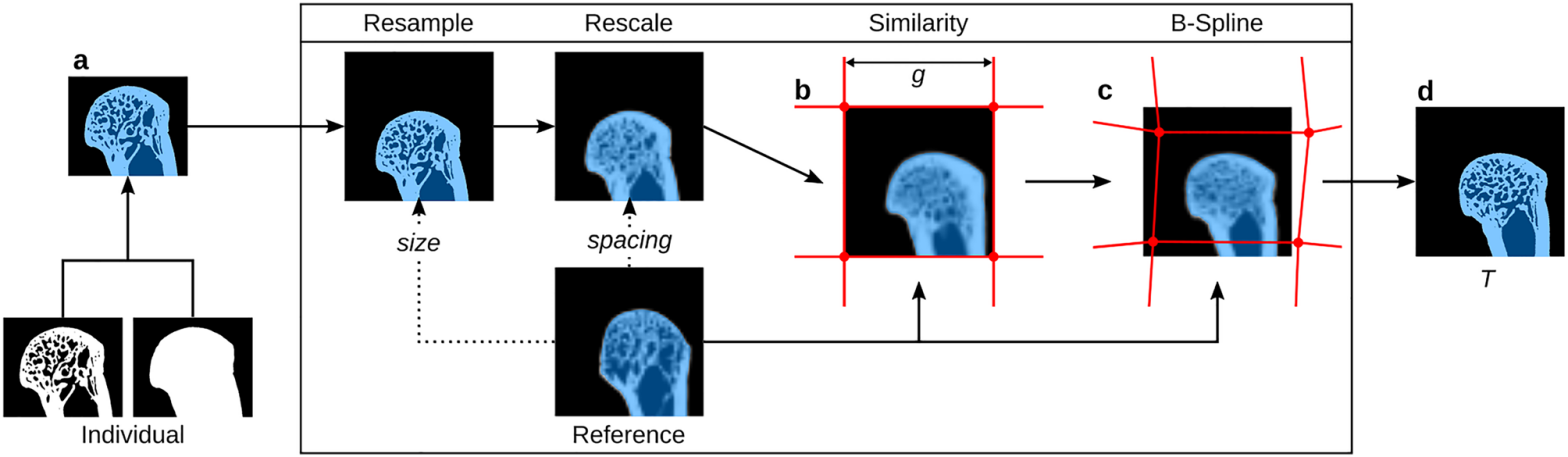
Schematic overview of a single registration run in 2D, which is in principle similar to the real registration in 3D. Original binary segmented $\upmu$CT image and mask are combined into masked image (**a**). This image is then resampled to the same size and rescaled to match the voxel spacing of the reference. Image after similarity transform using the reference image and undeformed B-spline control point grid with grid spacing *g* (**b**). Only grid nodes in the vicinity of the image domain are shown. Image after B-spline transformation with deformed control point grid (**c**). Transformed original image using the transformation *T* gained from the two registration steps (**d**).

Adaptations to the original workflow for QCT images are required for $\upmu$CT images because the runtime of the registration is proportional to the image size and hence registration of $\upmu$CT images can become impractical. Therefore, a resizing step was added to rescale the images to a resolution similar to that of QCT by increasing the voxel size by an isotropic factor. Rescaling the images before registration reduces the number of voxels and because the resulting transformations from the registration are smooth over the whole image domain, they can be applied to the original high-resolution images that accurately depict trabecular architecture as well^49^. The process of registration including the rescaling process is shown schematically in Fig. 3. However, the rescaling of the images also influences the registration quality, as with increasing scale factors, details are lost. Another parameter which influences the registration quality but also the overall runtime is the B-spline control grid spacing. A lower grid spacing can be used to register local features better at the expense of higher runtime. Therefore, optimal values for these two parameters have to be identified which allow for a feasible runtime with adequate registration quality.

Negative normalised cross correlation was used for both similarity and B-spline registration as cost-function for the optimisation. A conjugate gradient line search optimiser was used for the similarity and a L-BFGS-B optimiser^50^ for the B-spline registration. All other parameters were used as described by Steiner et al.^47^.

#### Mesh morphing

After the canonical bone image is created, each image is registered onto the canonical bone image. This yields transformations that are used to morph a canonical mesh, generated on the canonical bone, onto each individual bone. In this step, only the vertices of the meshes are moved in space, without altering their connectivity. Thus, isotopological meshes are created, that is, the topology of the meshes does not change. However, in this process the elements might get distorted, which can, in the extreme cases, lead to degraded elements. The degradation of the elements can be checked using various mesh metrics, which detect different types of degradation^51^. Because isotopological meshes are a requirement for the cHMA workflow, common strategies to repair degraded elements after morphing, such as local or global remeshing, can not be applied. Therefore, excessive mesh degradation has to be avoided in the first place. Hence, the registration procedure should yield transformations, which add minimal mesh distortion during the morphing step.

### Parameter identification

The registration method can be controlled by a set of parameters, which influence certain steps during the registration process. The cHMA workflow should allow analyses of high resolution ($\upmu$CT) scans in a reasonable amount of time, capture sufficient detail in the anatomical structure, but should avoid overfitting during the registration and distortions of the mesh.

To identify parameters suitable for morphometric analysis of $\upmu$CT images from bones of different species, a sample of thirty $\upmu$CT images of first metacarpals was used from a previous study^15^. The set contained ten samples each of *Homo sapiens*, *Pan paniscus*, and *Pan troglodytes verus*, respectively. The images had different voxel sizes ranging from 0.023 to 0.031 mm due to the usage of different scanners. Therefore, all images were resampled to an isotropic voxel size of 0.03 mm and a total image size of 674 $\times$ 681 $\times$ 1779 voxels during the registration procedure. All images were already segmented and masked from the previous study^15^. Images from the left hand were mirrored to match the right side. A rendering of all bones can be found in the Supplementary Material S1.

Two main parameters were varied in order to identify a suitable parameter set: (1) Image rescaling factor and (2) B-spline control grid spacing. The previously presented model by Steiner et al.^47^, developed for the proximal femur, used a grid spacing of 21 $\times$ 15 $\times$ 37 mm with an isotropic voxel size of 0.6 mm. Therefore, two different rescaling factors of 10 and 20 were tested, resulting in a voxel size of 0.3 mm and 0.6 mm respectively and the grid spacing was varied with 4, 6, 10, 20 and 30 mm distance. As the grid spacing is not the same in the three cardinal directions, the given spacings resulted in a grid of 5 $\times$ 5 $\times$ 13, 3 $\times$ 3 $\times$ 9, 2 $\times$ 2 $\times$ 5, 1 $\times$ 1 $\times$ 3, and 1 $\times$ 1 $\times$ 2 control points. A total of 10 different parameter runs were evaluated.

The full SDM workflow using two iterations, registration and mesh morphing was applied for each parameter run. Tetrahedral meshes were created using a characteristic edge length of 1 mm for all elements. The result was judged based on both image registration metrics and mesh distortion metrics as well as visual inspections of the registered images and morphed meshes. The overall runtime of the SDM workflow was measured for each parameter run. Image metrics, namely Dice coefficient, mean surface distance (MSD) and Hausdorff distance, were calculated between the transformed images and the canonical bone^52^. Mesh metrics, namely tet-collapse^53^ and volume-skew^54^ were calculated for the canonical and individual meshes. Briefly, tet-collapse measures the ratio of height and face area and is zero for a fully collapse tetrahedron and one for an optimal tetrahedron. Volume-skew measures the deviation in volume from an equilateral tetrahedron and is one for a degenerated tetrahedron and zero if the tetrahedron is equilateral. The final parameter set was selected by choosing the parameter set with the highest image metrics but lowest mesh degradation.

### Robustness

To evaluate robustness of the cHMA method, three different tests were performed: (1) the convergence behaviour of the SDM was investigated, (2) the influence of the starting images was evaluated and (3) a tenfold cross-validation was performed. All tests were performed using the chosen parameter set from the parameter identification and the metacarpal bone sample as described above.

In order to identify the number of iterations needed in the SDM, a model was created using a total of ten iterations, saving the results of intermediate iterations. The intermediate images were compared using the same image metrics as used for the parameter identification using the image of the final iteration as reference.

The bias towards the starting image was tested by generating a canonical bone with two iterations but choosing a different random starting image for each run. The canonical image of the parameter identification served as reference and image metrics were calculated using nine additional starting configurations.

Finally, a tenfold cross-validation was performed, i.e., leaving out three samples per run for the canonical bone creation. The same starting image was used, except for the run where this image was excluded, to get comparable results without the influence of the starting image. Again, two iterations were used in the SDM and the final canonical bone was compared using the image metrics to the canonical bone of the parameter identification.

### Case studies

To verify and showcase the applicability of the cHMA method, two case studies were performed on different bones. While the workflow can be applied using any morphometric quantity, only bone volume fraction was analysed for these case studies. Results were compared with previous studies to confirm that the same patterns can be observed with the new workflow.

#### First metacarpals

The first metacarpal data set, already described in the sections above, was used to replicate findings from a previous study on the same data set^15^. Specifically, Dunmore et al.^15^ reported higher relative BVTV (rBVTV) in the radiopalmar aspects of the head and base in *Homo* compared to the great apes. These results were based on qualitative interpretation of the volumetric distribution of trabecular morphometry, while quantitative assessment was limited to subcortical bone below the joint surfaces.

To test if cHMA can replicate and quantify these results for the entire volume, the workflow was run with the parameters as identified following the above sections. The canonical bone was created using two iterations in the SDM and the mesh was created using a characteristic element side length of 1 mm and morphed onto all bones. A background grid of 2.5 mm was used in the HMA process, with a sampling sphere diameter of 5 mm. BVTV was evaluated for all samples with HMA and rBVTV was calculated by normalising each sample’s BVTV by its mean BVTV. This step was necessary to compensate for systematic differences between taxa and allowed for the analysis of bone volume distribution while controlling for magnitude^16,23^.

Element-wise statistical tests were performed on the resulting rBVTV values mapped onto the isotopological meshes. Pairwise independent samples t-tests were used, followed by permutation tests for family-wise error rate (FWER) control^55^, yielding a new t-value threshold for significance. The significance level was set to $\alpha =0.05$. Moreover, principal component analysis (PCA) was calculated on the mesh elements to identify common and distinct rBVTV patterns among species. All values are given as mean $\pm$ standard deviation, if not denoted otherwise.

#### Proximal femur

A second case study was performed in order to check whether the results of a study on a larger bone, not part of the parameter identification procedure, can also be replicated. Thirty proximal femora, cut below the lesser trochanter, from a previous study were used^18,19^. The set contained ten samples of *Homo sapiens*, *Gorilla gorilla*, and *Pan troglodytes*, respectively. All images were already segmented and masked similar to the first metacarpals. A rendering of all bones can be found in the Supplementary Material S2.

Georgiou et al.^18^ found similar BVTV patterns for *Pan* and *Gorilla* while *Homo* showed a different pattern, lacking an anterior concentration in the femoral head. Moreover, the species specific pillar structures inside the femoral head, likely stemming from postural differences between the species, should be investigated further^19^. These findings were so far only described qualitatively.

Overall, the same settings as for the metacarpal sample set were used for both SDM and HMA. Only the image rescaling and mesh element size were adapted to reduce the runtime as a result of the larger bone size. Original voxel sizes ranged from 0.05 to 0.07 mm and were all resampled to an isotropic voxel size of 0.06 mm, leading to an image size of 1247 $\times$ 1836 $\times$ 1916 voxels during the registration. The B-spline grid setting resulted in 4 $\times$ 5 $\times$ 6 grid points over the image domain and 10$\times$ rescaling resulted in a voxel size of 0.6 mm during registration. Meshes for HMA were created using a characteristic edge length of 3 mm to account for the larger size of the bones compared to the metacarpals^38^. Statistical analysis was similar to the metacarpal case study. However, in addition to the full bone analysis, the head region was cropped and analysed separately to be consistent with Georgiou et al.^18,19^. This included thresholding the rBVTV distribution of the femoral head at the 80-percentile to qualitatively identify the presence of the previously observed pillar structures.

### Hardware and software

The whole registration and SDM framework was implemented in SimpleITK 2.0^56^ using Python 3.7 (Python Software Foundation, https://www.python.org). All other steps were performed directly in Medtool 4.5 (Dr. Pahr Ingenieurs e.U., http://www.medtool.at). Meshing was done using CGAL 4.11^57^ in Medtool. Visualisations were created in ParaView 5.9 (Kitware, https://www.paraview.org). Statistical analysis was done with SciPy 1.2.3^58^ in Medtool.

All registration regarding the first metacarpal were run on a Dual Intel(R) Xeon(R) Gold 6144 @ 3.50 GHz using 16 cores in parallel. All registration regarding the proximal femur were run on a Dual Intel(R) Xeon(R) E5-2697 v3 @ 2.60 GHz using 28 cores in parallel.

## Results

### Parameter identification

**Figure 4:**
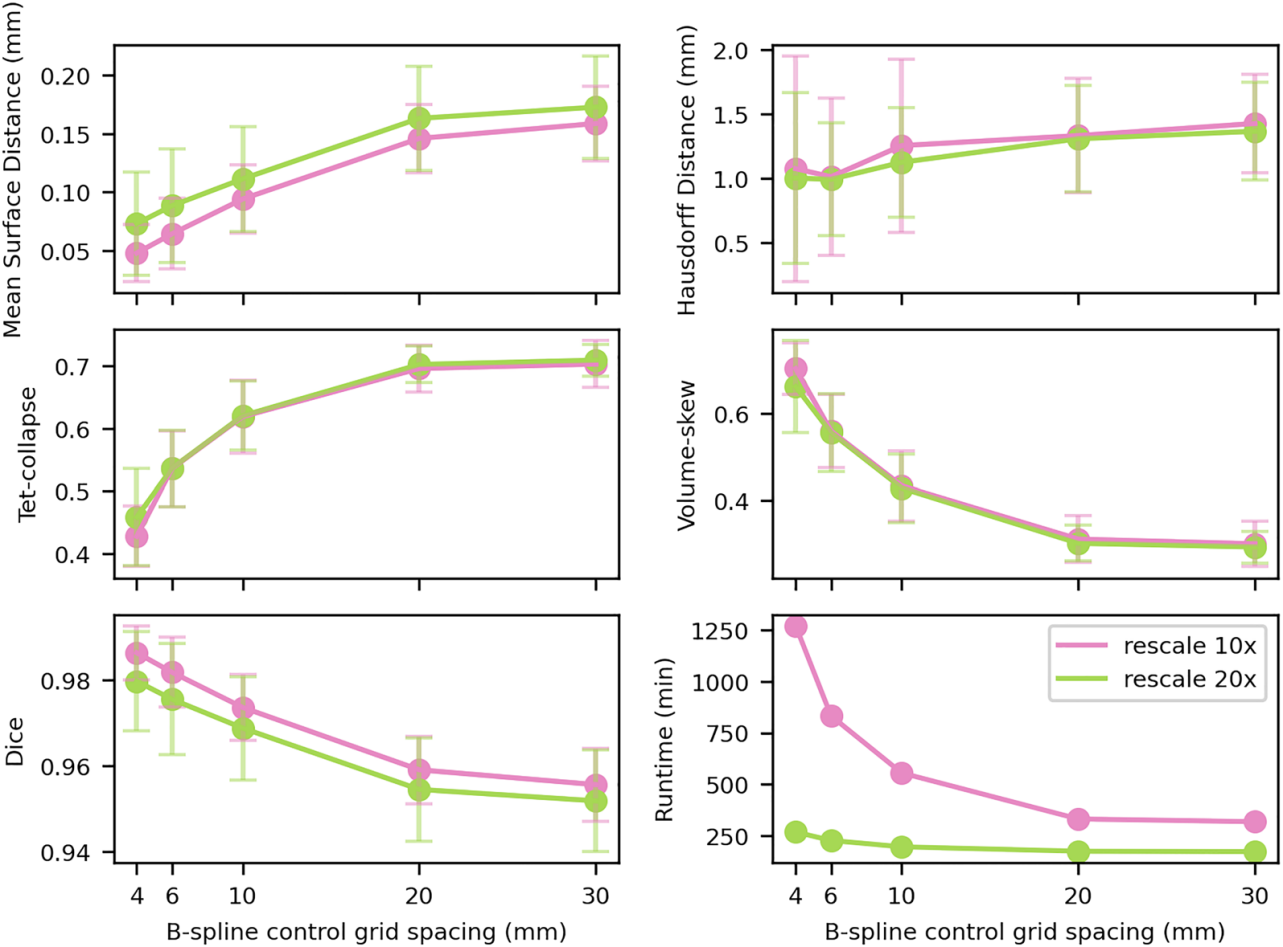
Average image and mesh metrics as well as runtime for the parameter identification studies. Each plot shows a single metric, split up for the two tested rescaling factors. The bars show the standard deviation. Runtimes are given for the canonical bone creation using 16 CPUs in parallel.

All ten parameter runs produced canonical bones and all bones were successfully registered onto it. Image and mesh metrics were calculated for each parameter run and the runtime for canonical bone creation and registration was tracked (Fig. 4). For larger B-spline grid spacings and higher rescaling factors, the runtime was lower than for small grid spacings and lower rescaling factors. The fastest run took 171 min, while the slowest run took 1270 min. Image metrics got better for lower grid spacings and lower rescaling factors, while mesh metrics got worse on average.

The mesh metrics showed a steep deterioration for grid spacings smaller than 20 mm, while the image metrics changed more gradually over the full range of tested spacings. As a result of this drastic change in mesh metrics, a 20 mm grid spacing was considered as the smallest possible grid spacing, without large mesh distortions. Single meshes had areas of distorted elements, apparent in the visual inspection, for grid spacings 10 mm and lower, while no distortions could be observed at 20 mm spacing or higher. The runtimes for 20 mm grid spacing were 330 min for 10$\times$ rescaling and 173 min for 20$\times$ rescaling. Despite longer runtimes, 10$\times$ rescaling was chosen as it had considerably better MSD on average (− 10%) with similar Hausdorff distance (+ 1.9%).

### Robustness

Using the identified parameters, a canonical bone was created using up to ten iterations. Qualitatively, no substantial change was visible for two or more iterations, suggesting that the model had converged (see Supplementary Fig. S1). Rigid body motion of the bone was observed, which resulted in a higher surface distance between two successive iterations than anticipated. The MSD between the final and penultimate iteration was 0.027 mm $\pm$ 0.026 mm with a Dice coefficient of 0.99. Two iterations were used for all further studies, as there was no visible change except for minor rigid body motion.

Qualitative inspection of the canonical bone with a different start image showed no considerable changes in bone shape but differences in bone size, translation and rotation (see Supplementary Fig. S3). The canonical bone size, ranged from 39 to 41.3 mm with a mean of 40.6 mm in the longitudinal axis. These size differences were also apparent in the MSD, which was 0.43 mm on average and ranged from 0.17 to 0.67 mm. Dice coefficient was 0.87 on average, ranging from 0.83 to 0.95.

All except one canonical bone in the cross-validation study were qualitatively similar in shape. This one canonical bone used a different starting image and thus resulted in a different sized bone, which is in line with the start image bias described above. MSD was 0.09 mm on average with values ranging from 0.04 to 0.34 mm and Dice coefficient was 0.97 on average, ranging from 0.91 to 0.99.

### Case studies

#### First metacarpals

All images were successfully registered onto the canonical bone. Dice coefficient was 0.96 $\pm$ 0.008, Hausdorff distance 1.33 mm $\pm$ 0.443 mm and MSD 0.146 mm $\pm$ 0.03 mm. The canonical mesh consisted of 10880 linear tetrahedral elements. Volume-skew for the canonical mesh was 0.49 $\pm$ 0.216 and tet-collapse 0.59 $\pm$ 0.153. After mesh morphing, the average volume-skew was 0.52 on average, ranging from 0.49 to 0.62 and tet-collapse was 0.56 on average, ranging from 0.5 to 0.58.

**Figure 5:**
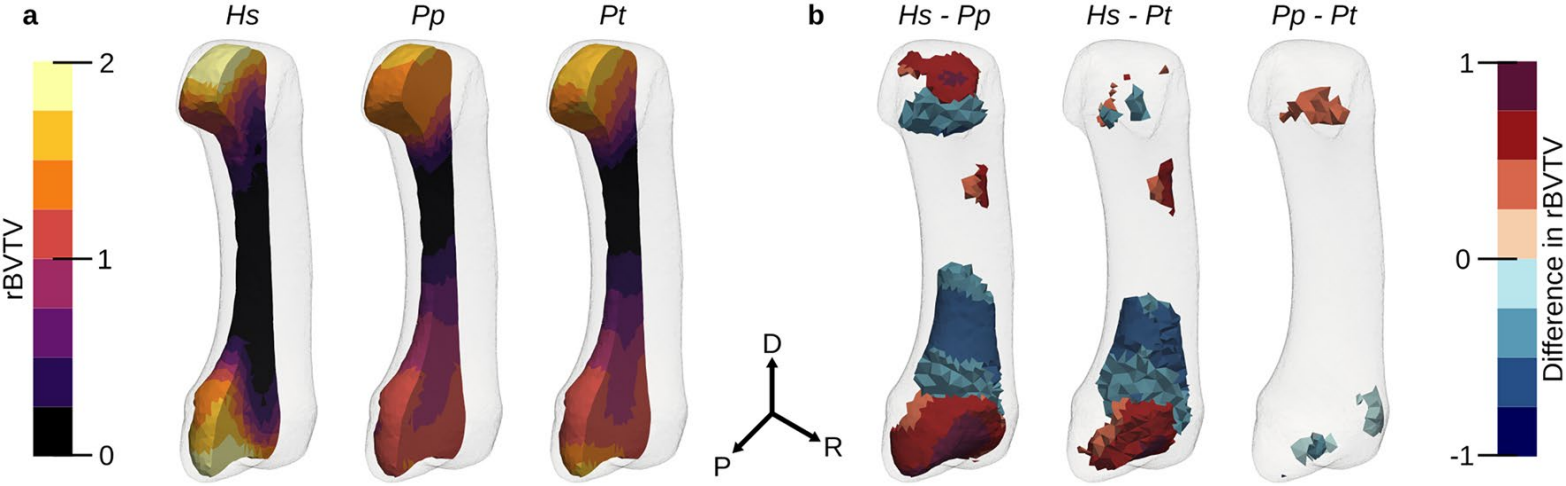
Average rBVTV and thresholded elements after FWER control. Average rBVTV per species cut along the sagittal plane (**a**). From left to right: *Hs* *Homo sapiens*, *Pp* *Pan paniscus*, *Pt* *Pan troglodytes*. Statistically significant groupwise differences, thresholded at critical level for $\alpha =0.05$ after FWER-control (**b**). Color gives the difference in rBVTV per species. For each pairwise comparison, a positive value (red) indicates that the first species has higher rBVTV while a negative value (blue) indicates that the second species has a higher rBVTV. *D* distal, *P* palmar, *R* radial.

**Figure 6:**
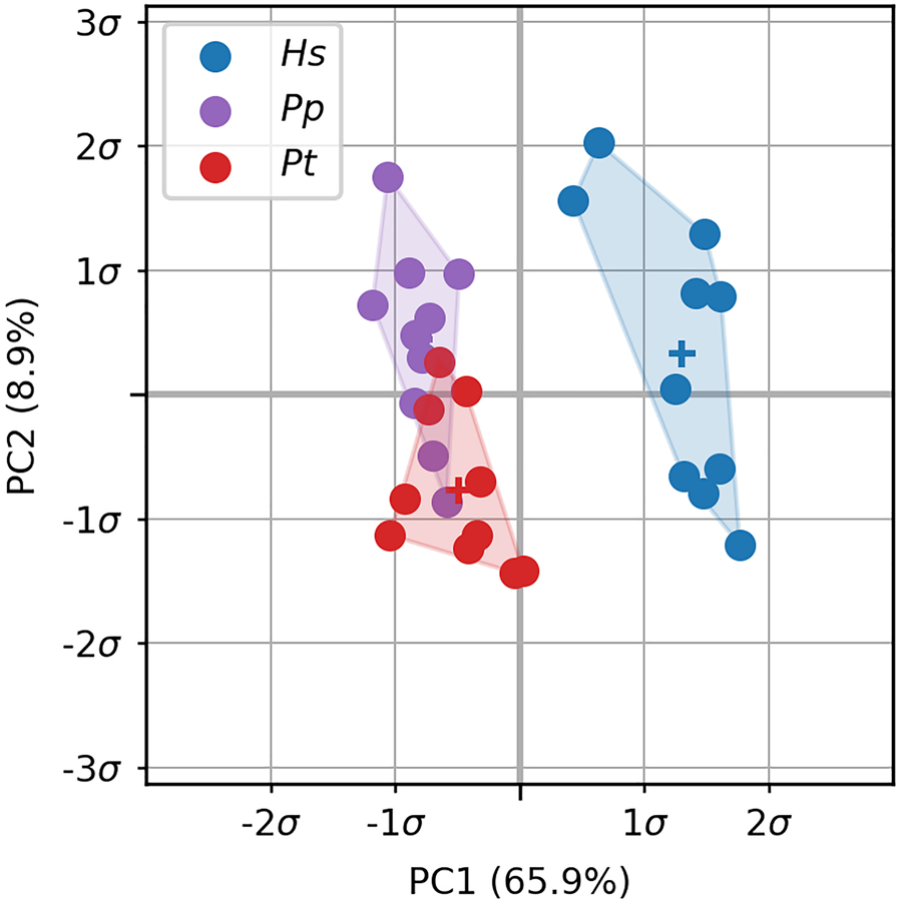
PCA plot of the first metacarpals. The plot shows the first two components in the principal component space for rBVTV. *Hs* *Homo sapiens*, *Pp* *Pan paniscus*, *Pt* *Pan troglodytes*.

Relative BVTV (rBVTV) was evaluated for all samples and group-wise comparisons between the species were done element wise. Mean rBVTV distributions for each species and thresholded canonical meshes for the three pairwise comparisons can be seen in Fig. 5. *Pan paniscus* and *Pan troglodytes verus* had similar rBVTV distributions, which can be seen in the low number of significant elements after family-wise error rate (FWER) control. However, both species significantly differed in comparison to *Homo*, which showed higher densities in the radio-palmar region in the base. A smaller area of significant difference in the radio-palmar aspect of the head can be seen in comparison to *Pan paniscus*. These results are in line with the previous study, however there are also differences found which were not visible in the previous study due to the limited focus on trabecular distribution immediately below the subchondral region. *Homo* shows a region of lower density in the proximal shaft region above the base, in comparison to *Pan*. There is also a smaller region of higher density in *Homo* in the disto-radial region at the shaft. In the PCA plot (Fig. 6) of the first two principal components of rBVTV, *Homo* separates well from *Pan* via the first component. The first component accounts for 65.9% of the total variance, and the second component for 8.9%.

#### Proximal femur

The canonical bone was successfully created and all femora were registered onto the canonical bone. The canonical mesh contained 13075 linear tetrahedral elements. Volume-skew for the canonical mesh was 0.25 $\pm$ 0.147 and tet-collapse 0.74 $\pm$ 0.12. After the mesh-morphing, volume-skew was 0.46 on average, ranging from 0.35 to 0.56 and tet-collapse was 0.6 on average, ranging from 0.53 to 0.67. Dice coefficient of the images after registration was 0.98 $\pm$ 0.005 and Hausdorff distance was 6.48 mm $\pm$ 4.022 mm, while the MSD was 0.246 mm $\pm$ 0.11 mm.

**Figure 7:**
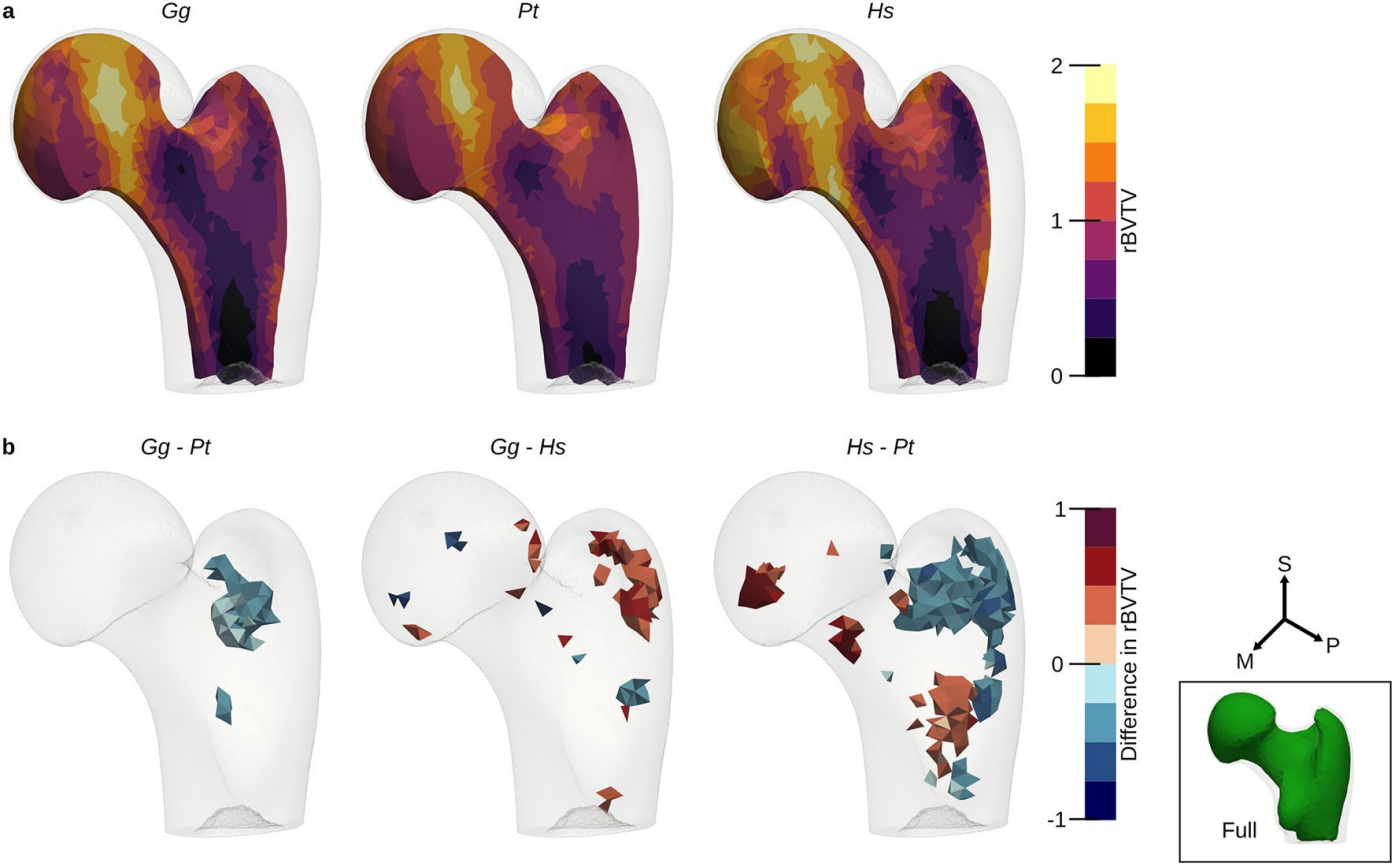
Mean rBVTV distribution (**a**) and statistical analysis after FWER control of the proximal femur (**b**). From left to right: *Gg* *Gorilla gorilla*, *Pt* *Pan troglodytes*, *Hs* *Homo sapiens*. *S* superior, *M* medial, *P* posterior.

**Figure 8:**
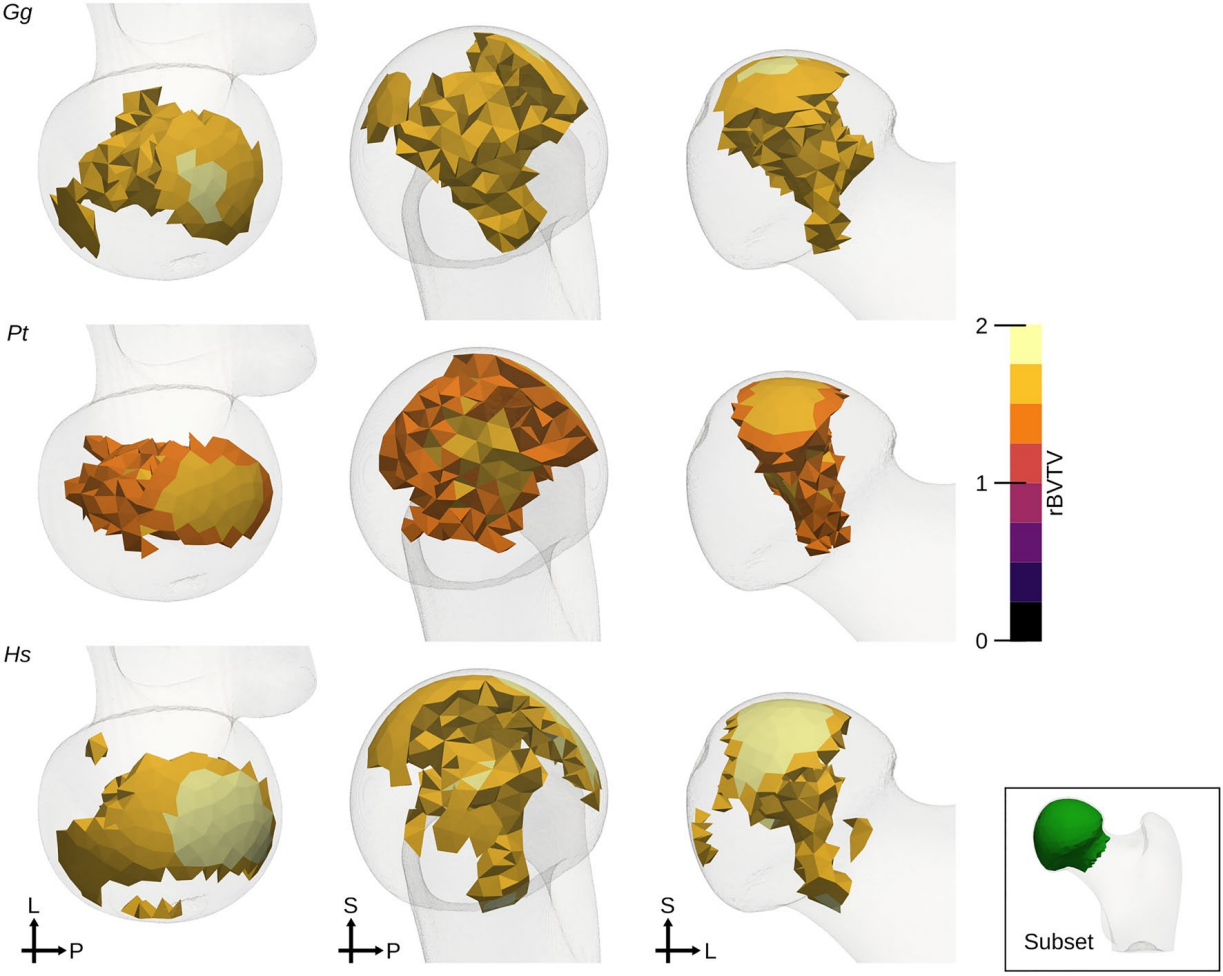
Thresholded femoral heads. rBVTV after thresholding at 80-percentile of each distribution in the femoral head only, shown here in three different views per species. From top to bottom: *Gg* *Gorilla gorilla*, *Pt* *Pan troglodytes*, *Hs* *Homo sapiens*. *L* lateral, *S* superior, *P* posterior.

**Figure 9:**
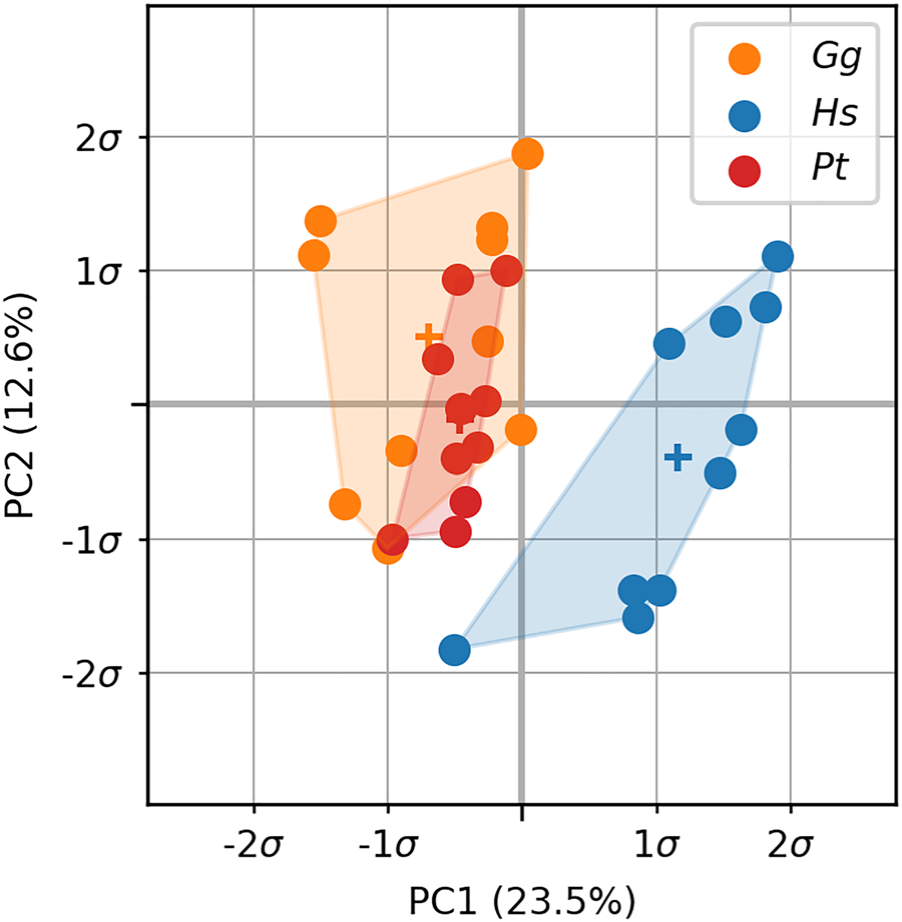
PCA plot for the femoral head. The plot shows the first two components in the principal component space for rBVTV. *Gg* *Gorilla gorilla*, *Hs* *Homo sapiens*, *Pt* *Pan troglodytes*.

The same HMA workflow was applied as for the first metacarpal bone. No clear pattern was visible in the univariate statistics for the full proximal femur (Fig. 7) after FWER control. Therefore, the elements in the head region were analysed separately. The head region, which consisted of 5140 elements, was extracted from the full mesh and rBVTV was calculated for this region separately. Less than 70 elements showed significant differences after FWER control in each pairwise comparison. The 80-percentile threshold of the rBVTV distribution of the mean mesh of each species showed a pillar structure inside the femoral head (Fig. 8). This pillar structure could be found in all three species and distally bifurcated into two pillars ending at the posterior and anterior aspects articular surface. The posterior pillar was present in all three species and extended anteriorly at the trabecular surface in only *Homo*. *Gorilla* and *Pan* exhibited an anterior pillar deep in the trabecular structure, which reached the trabecular surface in only *Gorilla*. The PCA plot (Fig. 9) for rBVTV in the head region showed a good separation of *Homo* versus *Gorilla* and *Pan*. However, the first principal component only accounted for 23.5% and the second for 12.6%.

## Discussion

The cHMA workflow shows the applicability of statistical deformation models to $\upmu$CT data, using a lower resolution image for the registration and the original, high-resolution image for the morphometric analysis with HMA. The method was able to replicate results from previous studies and also showed new, previously undetected differences between species. Furthermore, it was possible to confirm previously reported qualitative differences in a quantitative way. The canonical bone creation was robust and the registration produced images with high Dice coefficients and low MSD on average for both studied bones.

A similar method was recently presented by DeMars et al.^24^. The method is similar to the approach shown here, as HMA is also used to evaluate morphometric quantities. However, the meshes are created on the individual bones first and then correspondence is established using a coherent point drift algorithm. That means that meshes are created for all bones separately, while the here presented workflow uses isotopological meshes without the need of interpolation of values onto the canonical point cloud. Another similar workflow was presented by Taghizadeh et al.^59^. A canonical bone is constructed and meshed, however the individual meshes are then used to build a statistical model of shape, BVTV and anisotropy rather than doing element-wise statistical analysis. Another similar approach was used by Marangalou et al.^60^. However, in their method, a mesh-morphing approach with landmarks was used and not an SDM. All these three models were so far only applied to human bones. In contrast to other existing methods, the cHMA method was tested on bones of different species and provides a high level of automation. For instance, no landmarks need to be defined to run the model and $\upmu$CT data can directly be fed into the workflow.

Despite the high level of automation, the cHMA workflow still requires a number of parameters to be set manually. The parameter identification procedure highlighted the need to find a balance between capturing anatomical details and maintaining high quality meshes. Here, the parameters were identified by subjective criteria, i.e., minimal distortion in the meshes, maximal overlap of images, and visual inspection of the resulting meshes. A more objective measure would be beneficial in the future, however due to the lack of a gold-standard, it was not possible to select the parameters based on absolute measures. Except for the mesh size and voxel size during registration, the parameters identified from metacarpals could also be used for femoral bones. Even though the effective voxel size during registration was larger in the femora study than for the metacarpals, to reduce the runtime, the registration performance was still acceptable.

Although the method was generally robust, a marginal start image bias was observed that resulted in different location, rotation and scale of the canonical bone. This bias can be explained by the use of the similarity transform. The similarity transformation did not always produce images which would overlap in a true anatomical homologous position. For example, a rotation might also minimise the metric in the same way as scaling would do. To enforce a better initial alignment of the bones, anatomical landmarks could be used, similar to shape models. However, landmarking requires extra manual work and introduces new errors into the model. Another option would be the use of a different metric or tweaking the optimiser to find a better starting position.

The image registration worked in all cases, yielding high Dice coefficients above 95% on average and low MSD, less than the size of a single voxel in the rescaled versions (0.3 mm for the first metacarpals and 0.6 mm for the proximal femur). Hausdorff distances were slightly higher than expected, especially in the femur, with four individuals reaching distances above 10 mm. In particular, high surface distances were seen in the distal region of the femoral shaft. As the femora images were cropped at the shaft, it is likely that the high surface distance stem from those regions, similar to other studies where cut sections of bones were used^61^. However, these parts of the proximal femur are of less interest for trabecular analysis, as they contain almost no trabecular bone. Therefore, it is possible to remove parts of the bone from the analysis, by removing parts of the canonical bone before mesh generation or cropping the mesh afterwards.

Mesh morphing resulted in only minor deterioration of the mesh quality metrics, but individual elements in the final morphed meshes were still strongly distorted. This might be caused by the initial tetrahedral quality, which already showed some distorted elements. However, only a maximum of 0.12% of the elements failed for volume-skew metric (volume-skew larger than 0.9), and only up to 0.06% of the elements failed for aspect ratio, a metric commonly used in finite element modelling, for which an acceptable region is lower than 5% of all elements^62^. Furthermore, mesh quality is not a pressing issue here, as the models are not used for finite element analysis (FEA). When using mesh-morphing to create FE meshes, the initial mesh quality should be good, as deformation usually leads to distorted elements^63^. For further studies the canonical mesh quality should therefore be improved, to produce better morphed meshes. Other automatic meshing tools might yield better results and existing meshes could be re-meshed for better quality. However, even with a perfect canonical mesh, there is the chance that morphed meshes deteriorate, which can only be controlled for by using an optimiser procedure which incorporates information about mesh distortion, i.e., some sort of regularisation.

It was possible to run the full workflow on both metacarpal as well as femoral bones using the identified parameters. In both case studies, known differences between species could be found, and new, previously unreported differences in the trabecular bone could be observed. These novel significant differences were located in the trabecular bone, farther away from the cortical surface, which was previously not analysed. For the metacarpal bones, the spatial extent of differences at the base between *Homo* and *Pan* could be observed, including the density differences in the medullary cavity. Density patterns for the femoral head were consistent with previous results^18,19^. However, there was no significant difference in the full proximal femur or the femoral head using univariate statistics and the pillar structure was not as prominent in the averaged images as it was shown to be in individuals. Only multivariate statistics, using PCA, on the cropped femoral head demonstrated the previously seen difference between the species. These findings suggest that the pillar structure might be more variable between individuals and thus is not found in the averaged images. Hence, element-wise comparisons are not able to pick up the signal because the variance for this small sample set is too high.

This study has some limitations. Only a small subset of all available parameters was tested in a parameter study. All other parameters were used from literature. Additional parameter tuning could be done individually for each investigated bone to further improve the accuracy of the cHMA workflow. There was no regularisation method added in the B-spline registration, leading to distortions on the resulting meshes if the grid spacing was too small. The large B-spline grid spacing, used here, already regularised the deformation field to some extent, but other methods are available to gain more control over the B-spline, for example the imposition of bounds on the control grid displacements or control of the smoothness or rigidity of the resulting deformation field^64^. The creation of the canonical bone can be time-consuming, especially if lower B-spline grid spacing is used. Methods to further improve runtimes include using a B-spline pseudo inversion^65^ or iterative shape averaging^66^ for canonical bone creation. The sample set in these studies was relatively small, with only ten individuals per species. Furthermore, there was no differentiation regarding sex and age for both case studies, thus effects which are caused by ageing or due to sexual dimorphism might also explain the variability within the species. Therefore it should be repeated with a larger sample set and potentially also further separation of sexes and age groups. Additionally, all bones used in this study were not damaged or pathological, and high-quality CT-scans of them were available. Caution is warranted when the trabecular structure of potentially taphonomicaly altered bones is analysed in general^67,68^, and these cases could require adaptations to the cHMA method. FWER control was done using permutation tests, while random field theory (RFT) offers a faster, parametric approach^69,70^. However, RFT implementation for irregular tetrahedral meshes is challenging, and the requirements for the use of RFT might not be met by the data. Moreover, RFT currently does not support the use of non-parametric tests.

The here presented cHMA method shows an extension to the existing HMA method to quantitatively compare the trabecular morphology of bones of different species using a canonical bone and isotopological meshes. It works independent of the size of bones, is robust with respect to the choice of samples and starting image and the canonical bone creation converges quickly. The method can be applied directly to existing $\upmu$CT data sets and only requires segmented and masked images; it does not require landmarks. While the method was currently only tested on very well conserved bones of recent species, it shall be used for fossils as well in the future, where taphonomic damages pose an extra challenge in the registration and trabecular analysis. Furthermore, the cHMA workflow shall be applied to other bones, using more morphometric quantities and more samples to investigate also subtle differences and the local influence of morphometric quantities other than relative bone volume fraction.

## Supplementary Information

Supplementary Figures.

## References

[1] Ruff C, Holt B, Trinkaus E (2006). Who’s afraid of the big bad Wolff?: “Wolff’s law” and bone functional adaptation. Am. J. Phys. Anthropol..

[2] Seeman E (2009). Bone modeling and remodeling. Crit. Rev. Eukaryot. Gene Expr..

[3] Carlson KJ, Judex S (2007). Increased non-linear locomotion alters diaphyseal bone shape. J. Exp. Biol..

[4] Byron CD, Herrel A, Pauwels E, Muynck AD, Patel BA (2015). Mouse hallucal metatarsal cross-sectional geometry in a simulated fine branch niche. J. Morphol..

[5] Turcotte CM, Rabey KN, Green DJ, McFarlin SC (2022). Muscle attachment sites and behavioral reconstruction: An experimental test of muscle-bone structural response to habitual activity. Am. J. Biol. Anthropol..

[6] Karakostis FA, Jeffery N, Harvati K (2019). Experimental proof that multivariate patterns among muscle attachments (entheses) can reflect repetitive muscle use. Sci. Rep..

[7] Pontzer H (2006). Trabecular bone in the bird knee responds with high sensitivity to changes in load orientation. J. Exp. Biol..

[8] Barak MM, Lieberman DE, Hublin J-J (2011). A wolff in sheep’s clothing: Trabecular bone adaptation in response to changes in joint loading orientation. Bone.

[9] Currey JD (2011). The structure and mechanics of bone. J. Mater. Sci..

[10] Wallace IJ, Demes B, Judex S, Percival CJ, Richtsmeier JT (2017). Ontogenetic and genetic influences on bone’s responsiveness to mechanical signals. Building Bones: Bone Formation and Development in Anthropology.

[11] Tsegai ZJ, Skinner MM, Pahr DH, Hublin J-J, Kivell TL (2018). Systemic patterns of trabecular bone across the human and chimpanzee skeleton. J. Anat..

[12] Tsegai ZJ (2013). Trabecular bone structure correlates with hand posture and use in hominoids. PLoS ONE.

[13] Skinner MM (2015). Human-like hand use in *Australopithecus africanus*. Science.

[14] Stephens NB (2016). Trabecular architecture in the thumb of pan and homo: Implications for investigating hand use, loading, and hand preference in the fossil record. Am. J. Phys. Anthropol..

[15] Dunmore CJ, Bardo A, Skinner MM, Kivell TL (2019). Trabecular variation in the first metacarpal and manipulation in hominids. Am. J. Phys. Anthropol..

[16] Dunmore CJ, Kivell TL, Bardo A, Skinner MM (2019). Metacarpal trabecular bone varies with distinct hand-positions used in hominid locomotion. J. Anat..

[17] Tsegai Z, Skinner M, Pahr D, Hublin J-J, Kivell T (2018). Ontogeny and variability of trabecular bone in the chimpanzee humerus, femur and tibia. Am. J. Phys. Anthropol..

[18] Georgiou L, Kivell TL, Pahr DH, Buck LT, Skinner MM (2019). Trabecular architecture of the great ape and human femoral head. J. Anat..

[19] Georgiou L (2020). Evidence for habitual climbing in a pleistocene hominin in South Africa. Proc. Natl. Acad. Sci..

[20] Georgiou L, Kivell T, Pahr D, Skinner M (2018). Trabecular bone patterning in the hominoid distal femur. PeerJ.

[21] Colombo A (2019). Trabecular analysis of the distal radial metaphysis during the acquisition of crawling and bipedal walking in childhood: A preliminary study. Bull. et Mem. de la Soc. d’Anthropol. de Paris.

[22] Komza K, Skinner M (2019). First metatarsal trabecular bone structure in extant hominoids and swartkrans hominins. J. Hum. Evol..

[23] Sukhdeo S, Parsons J, Niu XM, Ryan TM (2018). Trabecular bone structure in the distal femur of humans, apes, and baboons. Anat. Rec..

[24] DeMars LJD (2020). Using point clouds to investigate the relationship between trabecular bone phenotype and behavior: An example utilizing the human calcaneus. Am. J. Hum. Biol..

[25] Judex S, Carlson KJ (2009). Is bone’s response to mechanical signals dominated by gravitational loading?. Med. Sci. Sports Exerc..

[26] Robling AG (2009). Is bone’s response to mechanical signals dominated by muscle forces?. Med. Sci. Sports Exerc..

[27] Kivell TL (2016). A review of trabecular bone functional adaptation: What have we learned from trabecular analyses in extant hominoids and what can we apply to fossils?. J. Anat..

[28] Bouxsein ML (2010). Guidelines for assessment of bone microstructure in rodents using micro-computed tomography. J. Bone Miner. Res..

[29] Maquer G, Musy SN, Wandel J, Gross T, Zysset PK (2015). Bone volume fraction and fabric anisotropy are better determinants of trabecular bone stiffness than other morphological variables. J. Bone Miner. Res..

[30] Kivell TL, Skinner MM, Lazenby R, Hublin J-J (2011). Methodological considerations for analyzing trabecular architecture: An example from the primate hand. J. Anat..

[31] Griffin NL (2010). Comparative forefoot trabecular bone architecture in extant hominids. J. Hum. Evol..

[32] Chirchir H, Zeininger A, Nakatsukasa M, Ketcham RA, Richmond BG (2017). Does trabecular bone structure within the metacarpal heads of primates vary with hand posture?. C.R. Palevol.

[33] Mueller TL (2009). Non-invasive bone competence analysis by high-resolution pqct: An in vitro reproducibility study on structural and mechanical properties at the human radius. Bone.

[34] Sode M, Burghardt AJ, Kazakia GJ, Link TM, Majumdar S (2010). Regional variations of gender-specific and age-related differences in trabecular bone structure of the distal radius and tibia. Bone.

[35] Rubinacci A (2011). Comparative high-resolution pqct analysis of femoral neck indicates different bone mass distribution in osteoporosis and osteoarthritis. Osteoporos. Int..

[36] Stephens NB, Kivell TL, Pahr DH, Hublin J-J, Skinner MM (2018). Trabecular bone patterning across the human hand. J. Hum. Evol..

[37] Du J (2019). Characterising variability and regional correlations of microstructure and mechanical competence of human tibial trabecular bone: An in-vivo hr-pqct study. Bone.

[38] Gross T, Kivell TL, Skinner MM, Nguyen N, Pahr DH (2014). A ct-image-based framework for the holistic analysis of cortical and trabecular bone morphology. Palaeontol. Electron..

[39] Sylvester AD, Terhune CE (2017). Trabecular mapping: Leveraging geometric morphometrics for analyses of trabecular structure. Am. J. Phys. Anthropol..

[40] Gunz P, Mitteroecker P (2013). Semilandmarks: A method for quantifying curves and surfaces. Hystrix Ital. J. Mammal..

[41] Myronenko A, Song X (2010). Point set registration: Coherent point drift. IEEE Trans. Pattern Anal. Mach. Intell..

[42] Rueckert, D., Frangi, A. F. & Schnabel, J. A. Automatic construction of 3d statistical deformation models using non-rigid registration. *In Medical Image Computing and Computer-Assisted Intervention—MICCAI*, Vol. 77–84. 10.1007/3-540-45468-3_10 (Springer, 2001).

[43] Bijar A, Rohan P-Y, Perrier P, Payan Y (2016). Atlas-based automatic generation of subject-specific finite element tongue meshes. Ann. Biomed. Eng..

[44] Pahr DH, Zysset PK (2009). From high-resolution ct data to finite element models: Development of an integrated modular framework. Comput. Methods Biomech. Biomed. Eng..

[45] Pahr DH, Zysset PK (2007). Influence of boundary conditions on computed apparent elastic properties of cancellous bone. Biomech. Model. Mechanobiol..

[46] Pahr DH, Zysset PK (2009). A comparison of enhanced continuum fe with micro fe models of human vertebral bodies. J. Biomech..

[47] Steiner L, Synek A, Pahr DH (2021). Femoral strength can be predicted from 2D projections using a 3D statistical deformation and texture model with finite element analysis. Med. Eng. Phys..

[48] Markley FL, Cheng Y, Crassidis JL, Oshman Y (2007). Averaging quaternions. J. Guid. Control. Dyn..

[49] Taghizadeh E (2016). Biomechanical role of bone anisotropy estimated on clinical CT scans by image registration. Ann. Biomed. Eng..

[50] Zhu C, Byrd RH, Lu P, Nocedal J (1997). L-bfgs-b: Fortran subroutines for large-scale bound-constrained optimization. ACM Trans. Math. Softw..

[51] Shewchuk, J. R. What is a good linear element? Interpolation, conditioning, and quality measures. In *Eleventh International Meshing Roundtable (Ithaca, New York)*, 115–126 (2002).

[52] Taha AA, Hanbury A (2015). Metrics for evaluating 3D medical image segmentation: Analysis, selection, and tool. BMC Med. Imaging.

[53] MSC Software. *Patran 2012 Reference Manual Part 3: Finite Element Modeling* (2012).

[54] Wang B, Mei G, Xu N (2020). Method for generating high-quality tetrahedral meshes of geological models by utilizing CGAL. MethodsX.

[55] Nichols T, Hayasaka S (2003). Controlling the familywise error rate in functional neuroimaging: A comparative review. Stat. Methods Med. Res..

[56] Lowekamp BC, Chen DT, Ibáñez L, Blezek D (2013). The design of SimpleITK. Front. Neuroinform..

[57] The CGAL Project (2017). CGAL User and Reference Manual.

[58] Virtanen P (2020). SciPy 1.0: Fundamental algorithms for scientific computing in python. Nat. Methods.

[59] Taghizadeh E, Chandran V, Reyes M, Zysset P, Büchler P (2017). Statistical analysis of the inter-individual variations of the bone shape, volume fraction and fabric and their correlations in the proximal femur. Bone.

[60] Marangalou JH, Ito K, Taddei F, van Rietbergen B (2014). Inter-individual variability of bone density and morphology distribution in the proximal femur and t12 vertebra. Bone.

[61] Joshi AA, Leahy RM, Badawi RD, Chaudhari AJ (2016). Registration-based morphometry for shape analysis of the bones of the human wrist. IEEE Trans. Med. Imaging.

[62] Burkhart TA, Andrews DM, Dunning CE (2013). Finite element modeling mesh quality, energy balance and validation methods: A review with recommendations associated with the modeling of bone tissue. J. Biomech..

[63] Grassi L (2011). Evaluation of the generality and accuracy of a new mesh morphing procedure for the human femur. Med. Eng. Phys..

[64] Rueckert D, Aljabar P, Paragios N, Duncan J, Ayache N (2015). Non-rigid registration using free-form deformations. Handbook of Biomedical Imaging.

[65] Yu W, Tannast M, Zheng G (2017). Non-rigid free-form 2d–3d registration using a b-spline-based statistical deformation model. Pattern Recogn..

[66] Rohlfing, T., Brandt, R., Maurer, C. & Menzel, R. Bee brains, B-splines and computational democracy: Generating an average shape atlas. In *Proc. IEEE Workshop on Mathematical Methods in Biomedical Image Analysis (MMBIA 2001)*, 187–194. 10.1109/MMBIA.2001.991733 (2001).

[67] Spoor, F., Jeffery, N. & Zonneveld, F. *Development, Growth and Evolution: Implications for the Study of the Hominid Skeleton, Chap. Imaging Skeletal Growth and Evolution. Linnean Society Symposium Series*, 1 edn, 123–162 (Academic Press, 2000).

[68] Bishop PJ, Clemente CJ, Hocknull SA, Barrett RS, Lloyd DG (2016). The effects of cracks on the quantification of the cancellous bone fabric tensor in fossil and archaeological specimens: A simulation study. J. Anat..

[69] Worsley K, Andermann M, Koulis T, MacDonald D, Evans A (1999). Detecting changes in nonisotropic images. Hum. Brain Mapp..

[70] Adler, R. J., Bartz, K., Kou, S. C. & Monod, A. *Estimating Thresholding Levels for Random Fields via Euler Characteristics*, Vol. 1704, 08562 (2017).

