# Supplementary material for: A computational framework for canonical holistic morphometric analysis of trabecular bone
Source: Sci Rep. 2022 Mar 25;12:5187. doi: 10.1038/s41598-022-09063-6 (PMC8956643; doi:10.1038/s41598-022-09063-6)
Supplement: Supplementary file 1 — Supplementary Figures. [file 41598_2022_9063_MOESM1_ESM.pdf]

Supporting information

S1

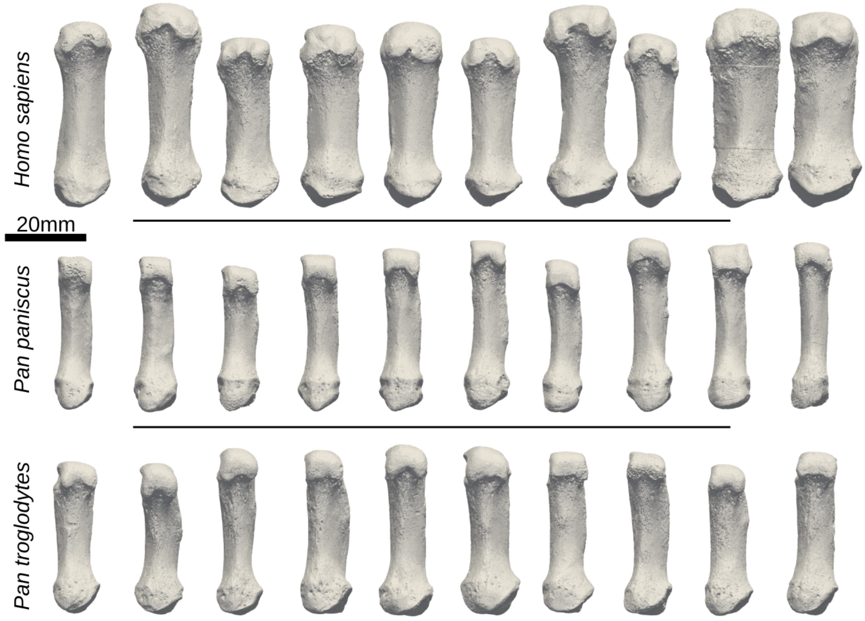

Figure 1: Overview of all first metacarpal samples in a frontal view.

S2

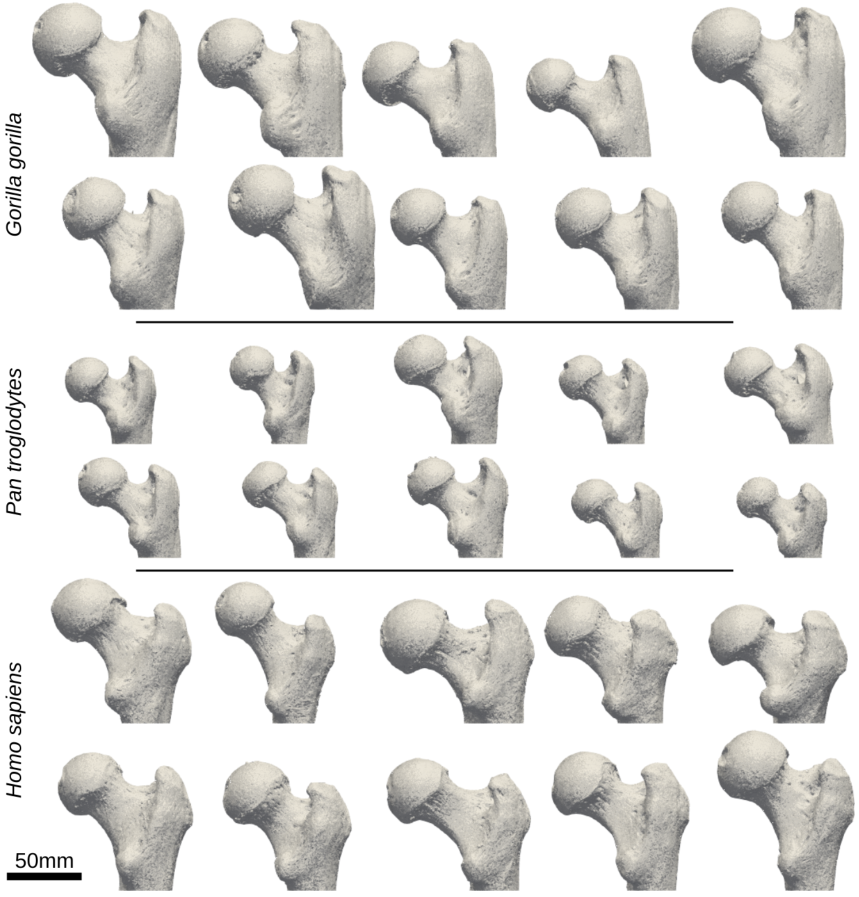

Figure 2: Overview of all femora samples in a frontal view.

S3

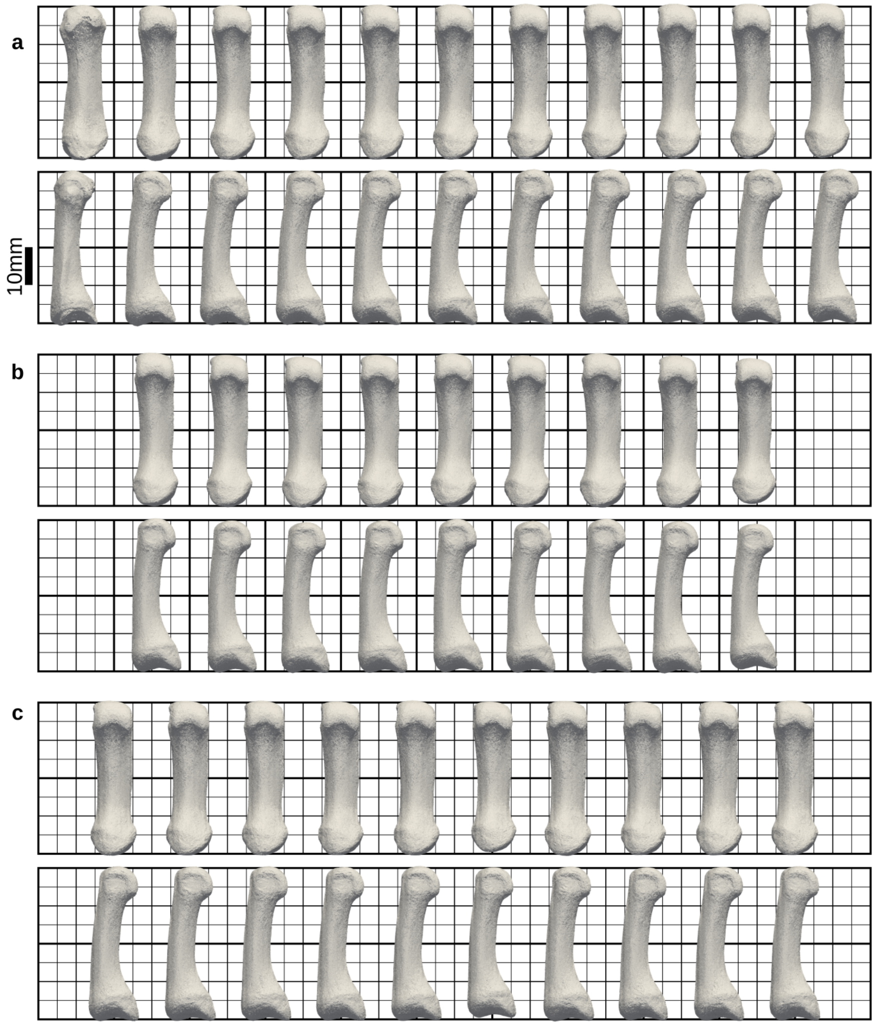

Figure 3: Overview over all robustness studies.
Ten iteration test (**a**). The first image is the initially chosen start image. For the start image bias test (**b**), differences in longitudinal height can be seen. Tenfold cross-validation (**c**). The sixth image had another start image than the other images.
